# Novel APC gene mutations associated with protein alteration in diffuse type gastric cancer

**DOI:** 10.1186/s12881-017-0427-2

**Published:** 2017-06-02

**Authors:** Souvik Ghatak, Payel Chakraborty, Sandeep Roy Sarkar, Biswajit Chowdhury, Arup Bhaumik, Nachimuthu Senthil Kumar

**Affiliations:** 1 Department of Biotechnology, Mizoram University, Aizawl, 796004 Mizoram India; 2 Department of Pathology, Agartala Government Medical College, Tripura, India

**Keywords:** Adenomatous polyposis coli (APC), Gastric adenocarcinomas, Immunoreactivity, Mutation, Cell cycle

## Abstract

**Background:**

The role of adenomatous polyposis coli (APC) gene in mitosis might be critical for regulation of genomic stability and chromosome segregation. APC gene mutations have been associated to have a role in colon cancer and since gastric and colon tumors share some common genetic lesions, it is relevant to investigate the role of APC tumor suppressor gene in gastric cancer.

**Methods:**

We investigated for somatic mutations in the Exons 14 and 15 of APC gene from 40 diffuse type gastric cancersamples. Rabbit polyclonal anti-APC antibody was used, which detects the wild-type APC protein and was recommended for detection of the respective protein in human tissues. Cell cycle analysis was done from tumor and adjacent normal tissue.

**Results:**

APC immunoreactivity showed positive expression of the protein in stages I, II, III and negative expression in Stages III and IV. Two novel deleterious variations (g.127576C > A, g.127583C > T) in exon 14 sequence were found to generate stop codon (Y622* and Q625*)in the tumor samples. Due to the generation of stop codon, the APC protein might be truncated and all the regulatory features could be lost which has led to the down-regulation of protein expression. Our results indicate that aneuploidy might occurdue to the codon 622 and 625 APC-driven gastric tumorigenesis, in agreement with our cell cycle analysis. The APC gene function in mitosis and chromosomal stability might be lost and G1 might be arrested with high quantity of DNA in the S phase. Six missense somatic mutations in tumor samples were detected in exon 15 A-B, twoof which showed pathological and disease causing effects based on SIFT, Polyphen2 and SNPs & GO score and were not previously reported in the literature or the public mutation databases.

**Conclusion:**

The two novel pathological somatic mutations (g.127576C > A, g.127583C > T) in exon 14 might be altering the protein expression leading to development of gastric cancer in the study population. Our study showed that mutations in the APC gene alter the protein expression and cell cycle regulation in diffuse type gastric adenocarcinoma.

**Electronic supplementary material:**

The online version of this article (doi:10.1186/s12881-017-0427-2) contains supplementary material, which is available to authorized users.

## Background

Gastric cancer is one of the most common cancer worldwide and there are more than 100 new cases per year in Tripura, Northeast India with a 5-year survival rate < 10% [1]. A number of genetic abnormalities have been identified in gastric cancer, including mutations in tumor suppressor gene [2].However, the abnormalities individually exhibit frequencies of less than 50% in gastric tumors, and are variable depending on the population and number of the samples analysed.

The human APC (adenomatous polyposis coli) gene is a tumor suppressor gene located on the long (q) arm of chromosome 5 and it encodes a protein of 312 kDa with 2843 amino acids. Inactivation of the APC geneis thought to be an initiating event for carcinogenesis [3]. Germline mutations of the APC gene are responsible for familial adenomatous polyposis (FAP) [4, 5]. About 700 mutations in the APC gene have been identified and most of these mutations lead to the production of short and abnormalprotein which cannot suppress the cellular overgrowth,leading to the formation of polyps and become cancerous [6]. The APC gene inhibits the members of Wnt signalling pathway that promotes β-catenin expression as a stimulator of cell division within the intestinal crypts [7]. A functioning APC protein is thus vital in maintaining low levels of cytosolic β-catenin, thereby preventing excessive cell proliferation [8]. APC controls metaphase-anaphase transition and mitotic exit and regulates G1 phase [9, 10]. Over-expression of APC in fibroblasts and colon cancer cell lines leads to arrest of G1 phase in the cell cycle [11, 12]. Role of APC in mitosis is critical for regulation of genomic stability and chromosome segregation [13]. Somatic mutations in the APC gene have been described in several tumour types such as pancreatic cancer [14], oral squamous-cell carcinoma [15] and oesophageal cancer [16]. APC mutations have been reported in gastric adenomas [17, 18] and in differentiated and signet-ring cell carcinomas [19]. Furthermore, frequent loss of heterozygosity on chromosome 5q has been detected in gastric carcinomas, particularly in well-differentiated type [20].Moreover, some differentiated types of gastric carcinoma are thought to originate from the intestinal metaplastic regions in gastric mucosa [21].

Since gastric and colon tumors share some common genetic lesions [22], it is relevant to investigate the role of APC tumor suppressor gene in the case of diffuse type gastric cancerwhich is not well characterized. Exons 14 and 15 are the most frequently mutated region for colorectal and gastric cancer as well as patients with familial adenomatous polyposis [23]. To clarify the role of APC gene mutations in the development of diffuse type gastric adenocarcinoma, we have investigated the mutations in the exons 14 and 15 of APC gene in a North East Indian population.

## Methods

### Subjects

The study design and data collection methods have been described in detail previously [24]. For this study, a total of 62 gastric cancer (GC) patients with or without a family history of cancer (median age 58 years; range 37–79) who received treatment between September 2012 and February 2014 at Agartala Govt. Medical College, Tripura,Northeast India and 40 healthy volunteers (median age 52 years; range 31–73) were recruited. Individuals less than age 45 were classified as younger and those age 45 and older were classified as older. From the 62 samples, 40 diffuse type gastric tumor samples were selected and the patients with gastric neoplasms other than adenocarcinoma (MALT lymphoma, stromal or carcinoid tumors), secondary or recurrent GC, previous history of other malignancies or refusal to participate were excluded. The healthy control samples wereage and sex adjusted, and selected from same ethnic group, free of any other chronic diseases, not having any record of gastritis and not pre-treated for any other type of cancer. The tumour and adjacent normal tissue of the patients were grossed properly by a trained histopathology technician followed by preparation of paraffin block. Histologic assessment of tumor type and grade were performed routinely on 4 to 5 μm thick hematoxyline & eosin stained sections of formalin-fixed paraffinembedded tumors according to the criteria outlined in the World Health Organization Classification of Tumors. After staining, the cytopathological data was obtained from microscopic observationsto confirm that all the adjacent normal tissues were devoid of tumor cells.The blood samples were collected by an experienced laboratory technician using vacu-puncture procedure. The peripheral blood samples of the patients were kept in EDTA rinsed microcentrifuge tubes and 50 μl of blood samples were processed for DNA isolation. Medical charts were reviewed to obtain information on cancer treatment, clinical stage, previous disease history and weight history. All participants gave written informed consent to the study protocol which was approved by the Ethical Committee of the Civil Hospital, Mizoram and Mizoram University, India (B.12018/1/13-CH(A)/IEC).The study protocol was also approved by the Institutional Review Board of all institutes involved in the study.

### Immunohistochemical analysis

For the immunohistochemical study, 4-μm histological fragments were obtainedfrom the tumor tissue and adjacent normal tissue of the cases and placed on glass slides pre-treated with poly L-lysine (Sigma Chemical Co, MO, USA). Initially, histological slides were placed in an oven at 60 °C for 24 hours to obtain better tissue adhesion and deparaffinization. Deparaffinization was performed in three xylene baths at room temperature for 15 min and placed in three baths of absolute ethanol baths for 1 min each. The slides were washed in running water for 5 min and submitted to heat induced antigen recovery by steam in a 10 mM citrate buffer solution with pH 6.0 for 30 min. After cooling for 20 min at room temperature, the slides were washed in running water for 5 min and endogenous peroxidase blocking was performed using a hydrogen peroxide solution at 3% in four baths of 5 min each. The slides were again washed in running water for 5 min and then washed with phosphate buffered saline (PBS) (pH 7.2–7.6) for 5 min.

Rabbit polyclonal anti-APC antibody(ab52223) (Abcam, Japan) was used, which detects the wild-type APC protein and is recommended for detection of the respective protein in human tissue. Incubation was carried out at aconcentration of 1:100 in a humidified chamber at 4 °C for at least 16–18 hours (overnight). Subsequently, after three washes in PBS at pH 7.2 – 7.6, the incubation was performed with the streptavidin-biotin peroxidase kit (LSAB, DakoCytomation, CA, USA) in a humidified chamber at room temperature for 30 min. This step was followed by washes with PBS at pH 7.2–7.6 and development with liquid DAB (Sigma Chemical Co, MO, USA) at room temperature for 5 min. After washing in running water for 3 min, counter-staining was performed with Harris hematoxylin for 1 min. The sections were dehydrated in three baths with absolute ethanol and three baths of xylene and then mounted using cover slips with Entellan resin (Sigma Chemical Co., MO, USA) for analysis by optical microscopy. As positive control, slides with histological sections previously demonstrated as being positive for these antibodies were used. A similar slide was used as a negative control, subtracting the primary antibody from the reaction [25]. Staining was recorded as either present or absent. Presence of staining was not rated according to the intensity of staining. Extent of staining was graded as: 0, 0–10% of cells positive; 1, 10–50% of cells positive; 2, greater than 50% of cells positive for APC. Staining was considered positive, if the extent of staining was graded as 2. Staining was considered reduced, if the extent was graded as 1 and 0.

### DNA extraction from the blood sample

The lymphocytes from patients’ blood and unaffected control blood were separated by lysing the RBCs using a hypotonic buffer (ammonium bicarbonate and ammonium chloride, Hi-media) with minimal lysing effect on lymphocytes. Three volumes of RBC lysis buffer were added to the blood sample, mixed by vortexing and inverting thoroughly for 5 min and centrifuged (Eppendorf 5415R, Germany) at 2,000 × g for 10 min. The lymphocytes were used for DNA extraction by modified protocol of Ghatak et al. [26].

### DNA extraction from the tissue samples

Deparaffinization was carried out by adding 1 ml of xylene to the tumor and adjacent normal tissue section in each microfuge tube, followed by vigorous vortexing for 10 mins. and centrifuged at 12000 rpm for 10 mins. The supernatant was discarded and the deparaffinization steps were repeated once again, followed by rehydration through subsequent washings with 100%, 90 and 70% absolute ethanol diluted in RNase free DEPC treated water, respectively. The deparaffinised tumor and adjusted normal tissue from the cases was used for the DNA extraction by the modified protocol of Ghatak et al. [27].

### PCR amplification of exons14 and 15AB of APC gene

PCR was performed with the DNA from tumor, adjacent normal tissue, patient’s blood and unaffected control blood samples. The APC gene exon 14 was amplified by PCR using primers Exon14-F (5’- ACATAGAAGTTAATGAGAGAC -3’) and Exon14-R (5’- TTGCTTACAATTAGGTCTTTTTGA G -3’). The primers were designed for known polymorphic sites by using the IDT primer quest software. Polymerase chain reaction (PCR) was carried out in 25 μl total reaction volume, each containing 100 ng of template DNA, 0.2 pM of each primer, 2.5 μl of 10X PCR buffer, 1.5 mM MgCl_2_, 200 mMdNTPs, and 1 U of Taq DNA polymerase (Fermentas, Germany). The reaction mixture was heated to 94 °C for 5 minutes, followed by 30 cycles each consisting of 40 sec denaturation at 94 °C, 40 sec annealing at 54 °C, 1 min of extension at 72 °C and a final 5 min extension at 72 °C. The APC exon 15A-B region was amplified by using Exon 15A-BF (5’- GGCAAGACCCAAACACATAATAG-3’) and 15A-BR (5’- GGAGATTTCGCTCCTGAAGAA -3’).The polymerase chain reaction (PCR) was carried out in 25 μl total reaction volume, each containing 100 ng of template DNA, 0.2 pM of each primer, 2.5 μl of 10X PCR buffer, 1.5 mM MgCl2, 200 mMdNTPs, and 1 unit of Taq DNA polymerase. The reaction mixture was heated to 94 °C for 5 minutes, followed by 35 cycles each consisting of 30 sec denaturation at 94 °C, 30 sec annealing at 59 °C, 1 min and 30 sec of extension at 72 °C and a final 7 min extension at 72 °C. The PCR amplification products (10 μl) was subjected to electrophoresis in a 1.2% agarose gel in 1X TAE buffer at 80 V for 30 min, stained with (0.5ug/ml) Ethidium Bromide and images were obtained in GBOX gel documentation system (UK). PCR products were purified with a Qiagen gel extraction kit (Qiaquick columns; Qiagen, Chatsworth, CA) and stored at -20 °C until sequenced using ABI 3500 Genetic Analyzer (Singapore) in Department of Biotechnology, Mizoram University, India.

### Cell cycle estimation

0.1 g of grossly gastric tumor and adjacent normal gastric mucosa tissue from the caseswere used for cell cycle analysis. Cells were harvested by mechanical dis-aggregation and fine-needle aspiration. Two separate aliquots of 6 × 10^6^ tumor cells were prepared for each sample. Pellets were incubated with 250 mL of 0.1% RNAse (Sigma, St Louis, MO, USA) and 50 mg/mL Propidium iodide (presence of Sodium citrate and TritonX-100) for 30 min at 37 °C and flow cytometric analysis was performed by Facs Canto and DIVA software (BD, Germany). Four distinct phases could be recognized in a proliferating cell population: the G1, S- (DNA synthesis phase), G2- and M-phase (mitosis). G2- and M-phase could not be discriminated because of the presence of identical DNA content [28]. The data obtained was analyzed using the ModFit LT software (DNA Modeling System) version 2.0 (Verity Software House, Inc.) and single parameter histograms were obtained.

### Single-strand conformation polymorphism (SSCP) analysis

The 5’ half of exon 15 (codons 654- 1700) of APC gene was amplified using primer set (15A-B). An aliquot of 0.75 μl of each PCR product from tumor and adjacent normal gastric mucosa tissue were diluted with an equal volume of water and mixed with 1.5 μ1 of 95% formamide. This mixture was denatured at 95 °C for 5 min, cooled on ice and 2 μl was used for loading on SSCP gel (8% non-denaturing polyacrylamide gels). SSCP Gels were pre-run at 400 V, 20 mA, 2 W, for 10 or 50 volt-hours (Vh). Electrophoresis was performed at 400 V, 20 mA, 2 W, for 200–300 Vh. Electrophoresis was carried out at either 4, 10, 15 or 20 °C depending on the optimal temperature for a given PCR fragment [29]. The gels were ethidium bromide stained, and gel documented using Syngen-G-BOX (USA).

### Sequence analysis

The samples exhibiting polymorphism and instability after SSCP analysis was taken for further sequencing and mutation analysis. All PCR products from the tumor, adjacent normal tissue, blood and unaffected control blood were sequenced from opposite directions to ensure reading accuracy. Sequences and chromatograms obtained were examined by chromas software version 2.13, DNA baser and align by BLAST [www.ncbi.nlm.nih.gov/blast]. The APC exons 14 and 15 were checked from Gene card database [HGNC - 583, Entrez Gene - 324, Ensembl - ENSG00000134982, OMIM - 611731, UniProtKB - P25054]. The sequences of tumor, adjacent normal was compared and sequence variation in tumor tissues from adjacent normal was recorded as somatic mutations. Further, it was confirmed that the sequence of patient’s adjacent normal, blood and healthy control blood samples are 100% identical. All the sequences containing the mutation were evaluated for their potential pathogenicity using the following algorithms: DNA baser version 3.5.4.2, Codon Code aligner version V.4.2.2, Mutation taster [www.mutationtaster.org/], PolyPhen-2 [http://genetics.bwh.harvard.edu/pph2/index.shtml.], SIFT [http://sift.jcvi.org], Mutation Assessor [http://mutationassessor.org/]. The MEGA Align algorithm was used at two depths of alignment [Cancer to Normal and Normal to database sequences]. The results of PolyPhen-2 was retrieved from the original webpage [version 2.2.2] but also from version 2.0.22 run by PON-P and version 1 run by Condel, which use them for weighted average scores. Circos plot [30] was generated to visualize the mutations in exons 14 – 15, protein expression and their association with gastric tumor stages and ploidy levels based on the observed data. This cross representation between mutations, APC protein expression and ploidy level explains the consequences of altered cell cycle regulation.

### Reconfirmation of mutations by restriction digestion

Codon 622 – 625 mutations in exons 14 alter the recognition site of restriction enzyme. The specific mutation detected together with restriction enzyme used and size of fragments expected after digestion of PCR products are given in Table 1. Digestion products were analysed by electrophoresis in 8% polyacrylamide gels which were stained with ethidium bromide and documented under UV light. Restriction digestion of PCR products was performed with the DNA from tumor, adjacent normal tissue and unaffected control blood samples.

**Table 1:** Somatic mutational profiling of APC gene exon 14 using PCR-RFLP

Codon	Enzymes	Size of normal alleles (bp)	Mutation	Amino acids	Size of mutant alleles	Sample Frequency
622^b^	*MspI*	189,163	TAC > TAA	Y > ^a^	352	10%
625^b^	*MaeI*	266, 86	CAG > TAG	Q > ^a^	135, 131, 86	5%

^a^represents stop codon

^b^represents Novel mutations (unreported in the database)

### Statistical analysis

Chi-square and Fisher’s exact tests were used to assess the association ofAPC protein expression and cell cycle distribution with APC gene mutation status in relation to the stage of gastric cancer. For all tests, a two-sided *P*-value <0.05 was considered statistically significant. All analyses were performed using R statistical package ver3.3.0 [31].

## Results

Gastric cancer was more prevalent in males (55%) in the Tripura population. The median age in the younger age group was 36 years (range16–45), and this group contained a lesser proportion of patients (35%) than the older age group (65%) (Table 2). The most common symptoms were abdominal pain followed by weight loss and vomiting in the case of older age patient group. Most of the gastric cancer patients were operated with stage II tumor. The symptoms at recruitment in both groups are shown in Table 2.

**Table 2:** Clinicopathological features of gastric cancer patients (Stratified by age)

Parameters	Younger age group (Age ≤ 45 years)	Older age group (Age 46–79 years)	*P*-value
Gender			
Male	06 (15%)	16 (40%)	0.326
Female	08 (20%)	10 (25%)	
BMI (Mean ± SD)	21.4 kg/m^2^ ± 3.6	22.1 kg/m^2^ ± 2.9	0.058
Tumor size (cm), (mean ± SD)	4.6 ± 2.8	4.9 ± 3.1	0.922
Tumor location			
Upper	08 (20%)	13 (32.5%)	0.869
Middle	02 (5%)	05 (12.5%)	
Lower	04 (10%)	06 (15%)	
Whole	0	02 (5%)	
Type of gastrectomy			0.186
Total	10 (25%)	12 (30%)	
Subtotal	04 (10%)	14 (35%)	
Stage			
Stage I	0	0	
Stage II	7 (17.5%)	12 (30%)	0.828
Stage III	5 (12.5%)	8 (20%)	
Stage IV	2 (5%)	6 (15%)	
Abdominal pain	9 (22.5%)	16 (40%)	0.161
Weight loss	5 (12.5%)	12 (30%)	0.05
Hemorrhage	7 (17.5%)	3 (7.5%)	0.205
Dysphagia	6 (15%)	5 (12.5%)	0.76
Early satiety	3 (7.5%)	3 (7.5%)	1.00
Vomiting	4 (10%)	12 (30%)	0.045
Increased Abdominal girth	1 (2.5%)	0	0.317

Values in parenthesis indicates percentage of that sample represented from the total number of studied samples

**Fig. 1:**
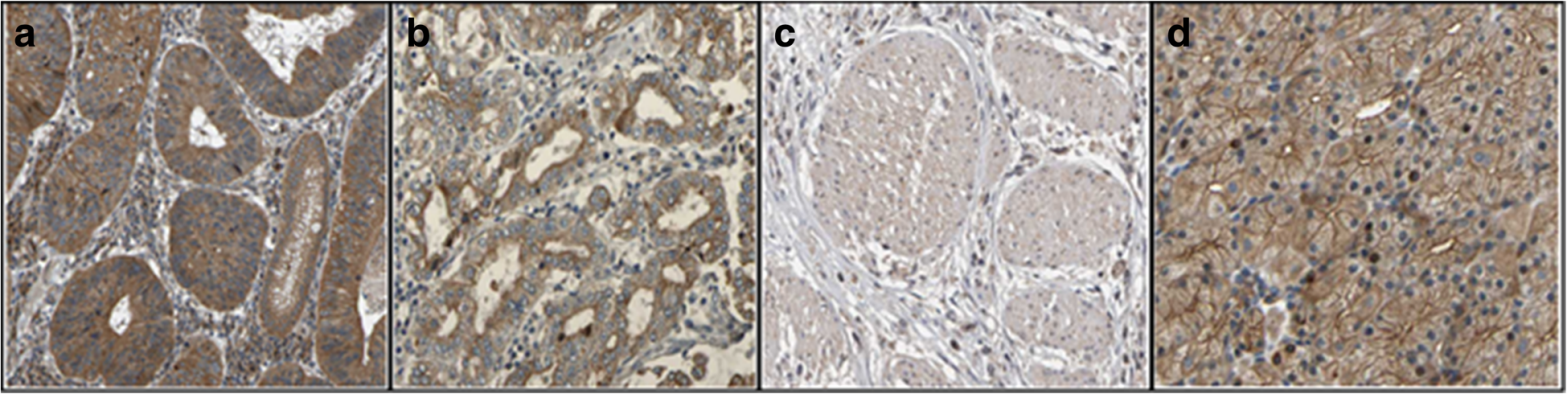
Microscopic view of well differentiated adenocarcinoma of gastric tumor cells. **a** Positive high immunoexpression of anti-APC antibody in cancer cell (**b**) Positive moderate immunoexpression of anti-APC antibody in cancer cell (**c**) Negative immunoexpression of anti APC antibody in cancer cell (**d**) Positive moderate immunoexpression of anti-APC antibody in adjacent normal cell (from negative immunoexpression cancer cell), represented by the brownish colour in the cytoplasm and membrane

The tumor samples used in the present study were diffuse type gastric adenocarcinoma as confirmed after H&E staining. Our data showed that 47.5% samples were in stage II, 32.5% in stage III and 20% in stage IV. In the normal control gastric mucosa, APC immunoreactivity was positive in all the 40 samples examined. Rabbit polyclonal anti-APC antibody (ab52223) (Abcam, Japan) specificity was reported for endogenous levels of total APC protein and is expressed in a variety of tissues (http://www.abcam.com/apc-antibody-ab52223.html).Maximal APC immunoreactivity was present in the cytoplasm of the cell, but staining was not present in the mucus vacuoles. In 10% of the adenocarcinoma sample, APC immunoreactivity was completely absent despite the abundant expression of the protein in the adjacent normal mucosa. Four samples (10%) were negative for APCprotein expression in adenocarcinoma and 36 (90%) werepositive (Table 3, Fig. 1).In gastric tumour Stage III, 7.5% of the samples showed negative protein expression.After performing the Fisher exact test, the APC expression was not significantlycorrelated with the Stages of gastric cancer (*p* = 0.077).APC immunoreactivity showedpositive expression of the protein in the stage I (47.5%), stage II (25%) and stage III (17.5%) gastric adenocarcinoma and Stage III (7.5%) and stage IV (2.5%) showed negative expression of the protein.

**Table 3:** Immunohistochemical staining of APC protein in different gastric cancer stages

Tissue Type	APC immunohistochemistry	
	Positive	Negative
Adjacent Normal cell	40 (100%)	0
Tumor cell		
Stage II	19 (47.5%)	0
Stage III	10 (25%)	3 (7.5%)
Stage IV	7 (17.5%)	1 (2.5%)

*P* value = 0.07

Values in parenthesis indicates percentage of that sample represented from the total number of samples

**Fig. 2:**
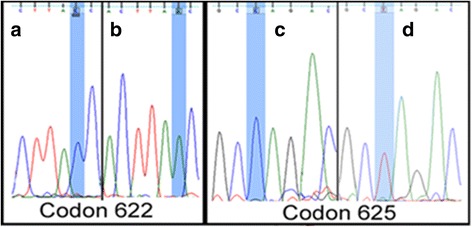
Different Mutation in the exon 14 (g.127576C > A, g.127583C > T) of APC protein. **a** Wild type codon 622 (TAC) in adjacent normal sample, **b** Mutant type codon 622 (TAA) in tumor sample, **c** Wild type codon 625 (CAG) in adjacent normal sample, **d** Mutant type codon (TAG) in tumor sample

**Fig. 3:**
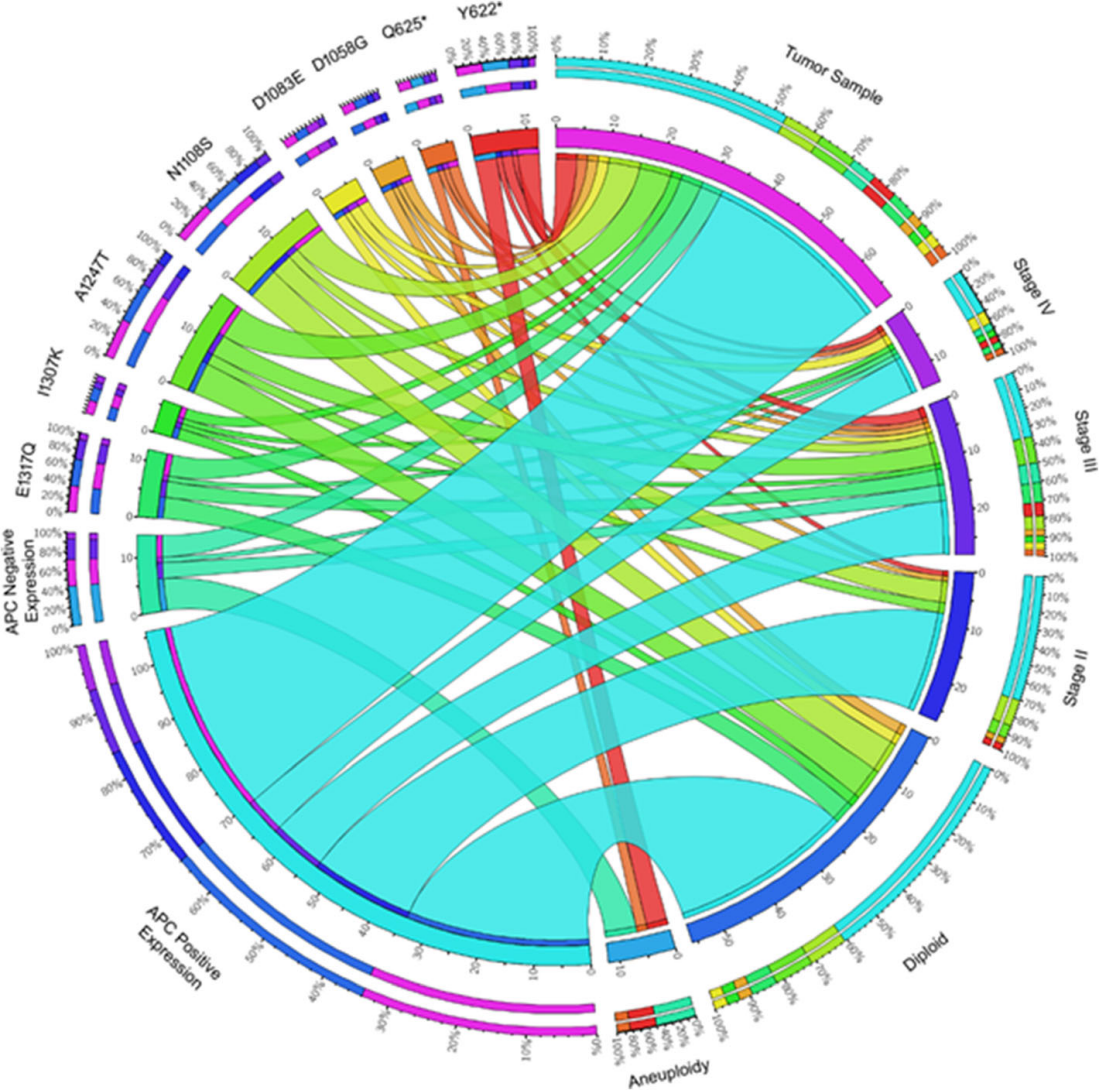
Circos plot of representative APC mutation in gastric tumor sample and their association with cancer stages, cell cycle, and APC protein expression. The frequency of occurrence of different factors such as mutations, APC protein expression pattern, ploidy level and tumor stages is depicted in the outer ring. The inner ring of circos plot depicts the association between the mutations, APC protein expression pattern, ploidy level and tumor stage involved in gastric cancer. Each factor has been assigned a color. The arc originates from mutations and APC protein expression status and terminates at tumor staging and ploidy level to compare the association between the origin and terminating factors. The area of each colored ribbon depicts the frequency of the samples related with the particular mutations and APC protein expression

We analysed the complete 352 bp coding region of exon 14 in the APC gene and found two novel deleterious sequence variations (g.127576C > A, g.127583C > T) changing the codons 622 and 625 to stop codons (Y622* and Q625*) in 10% of tumor samples. But, thesesomatic mutations were not observed in adjacent normal tissues and blood samples of patients as well as in healthy control blood samples (Table 1, Figs. 2 and 3). The mutation was reconfirmed at codons 622 and 625 by performing restriction digestion with *MspI* and *MsaI* (Additional file 1: Figure S1A). The wild type 622 codon (TAC) produced two digested products (189 bp and 163 bp), whereas mutant type codon (TAA) showed an uncut 352 bp band after *MspI* digestion. And, the 625 wild type codon (CAG) produced two digested products (266 bp and 86 bp), whereas mutant type codon (TAG) showed three distinct digested band (135 bp, 131 bp, 86 bp) in the polyacrylamide gel.

**Fig. 4:**
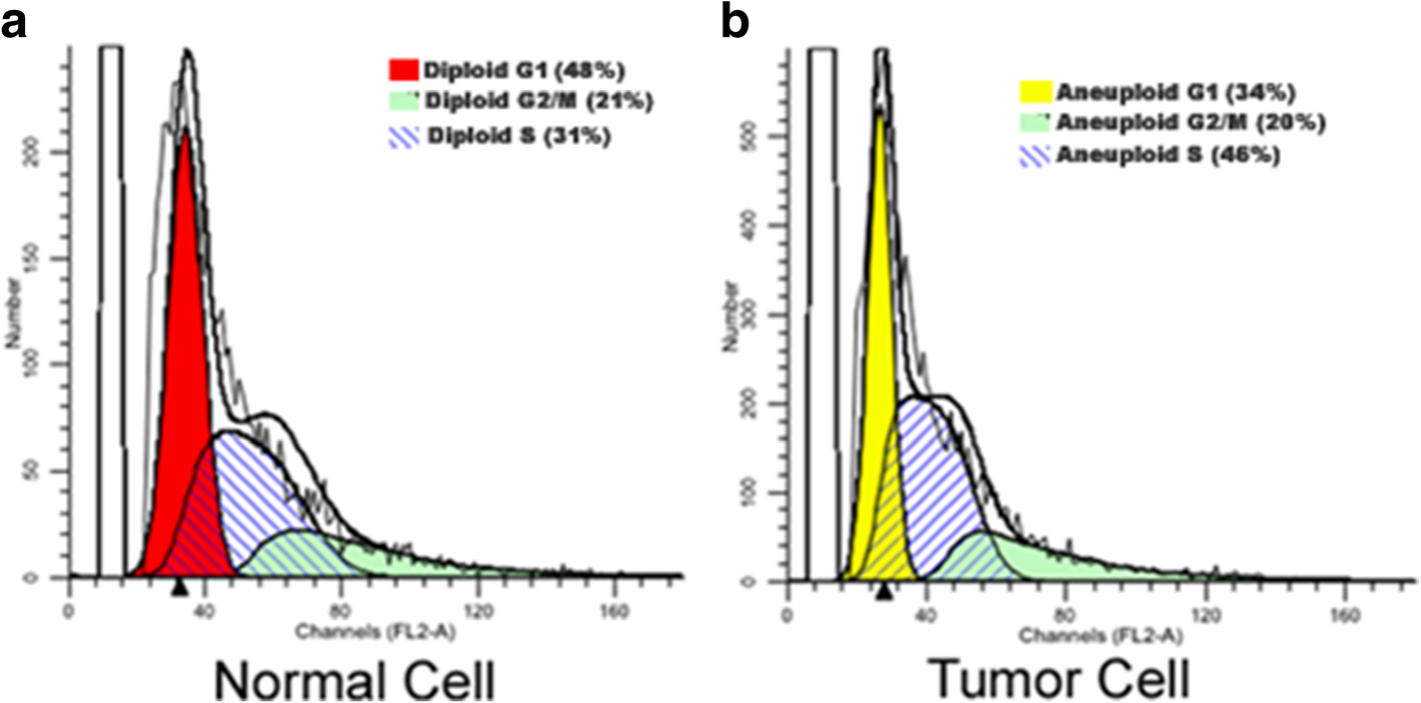
Histogram of Cell cycle analysis of (**a**) adjacent control gastric cell and (**b**) Tumor gastric adenocarcinoma cell (*p* value = 0.071)

Samples containing mutations in codon 622 and codon 625 ofexon 14 showed abnormal cell cycle stages and indicated that aneuploidy occurs due to Apc-driven gastric tumorigenesis. Samples with well differentiated diffuse type gastric adenocarcinoma showed a nonsense mutation from TAC (Y) to TAA (stop codon) at codon 622 and samples with poorly differentiated diffuse type gastric adenocarcinoma had a change from CAG (tryptophan) to TAG (stop codon) at codon 625 (Fig. 2) resulting in a truncated gene product. The tumor samples with Y622* and Q625* mutations exhibited G1 phase arrest with high S phase DNA (*p* value = 0.071) leading to loss of the role of APC in mitosis and chromosome stability [32].Most of the gastric cancer samples showed diploidy, except in samples containing 622 and 625 codon change where aneuploidy resulted in less DNA content in G2/M phase and high DNA content in S phase (Fig. 4).

The 936 bp coding region of exon 15 in the APC gene and somatic variants were detected in the gastric cancersamples (Table 4). We observed a change of exon 15 A-B region by SSCP (Additional file 1: Figure S1B). These tumour samples showedan instability banding pattern,unlike thematched adjacent normal tissue and blood of the patient’s sample as well as the healthy control blood samples. Further, these samples were sequenced and six missense somatic mutations (g.131270A > G, AA1058D > G; g.131346 T > G, AA1083D > E; g.131420A > G, AA1108N > S; g.131836G > A, AA1247A > T; g.132017 T > A, AA1307I > K; g.132046G > C, AA1317E > Q) were detected randomly in a total of 40% of tumor samples which causes abnormal protein products(Table 4, Fig. 3, Additional file 1: Figure S2). Among the six missense mutations, two (1058D > G and 1307I > K) were not previously reported in the literature or the public ensemble mutation databases. Both the mutations were pathological and disease causing based on SIFT, Polyphen2 and SNPs & GO scores. Most of the exon 15 mutations were found in the compositional bias region of the APC protein.

**Table 4:** Detection of somatic mutations in APC gene exon 15

Codon	Mutation	Amino acid change	Sift score	Polyphen2 score	SNPs & GO Effect/RI	Sample Frequency	Motifs	Domains	Amino acid property Change
1058	GAT > GGT^a^	Asp(D) > Gly(G)	Pathological	0.37 (Pathogenic)	Disease/3	5%	--	Beta‐Catenin Binding	• The charge of the wild-type residue will be lost, this can cause loss of interactions with other molecules or residues • The mutation introduces a more hydrophobic residue at this position. This can result in loss of hydrogen bonds and/or disturb correct folding.
1083	GAT > GAG	Asp(D) > Glu(E)	Natural	0.08 (Benign)	Natural/1	5%	--	--	• The mutant residue is bigger, this might lead to bumps.
1108	AAT > AGT	Asn(N) > Ser(S)	Natural	0.08 (Benign)	Disease/0	15%	--	Beta‐Catenin Binding	• The mutation introduces a more hydrophobic residue at this position. This can result in loss of hydrogen bonds and/or disturb correct folding.
1247	GCC > ACC	Ala(A) > Thr(T)	Natural	0.10 (Benign)	Natural/3	15%	GSK3 phosphorylation site	Beta‐Catenin Binding	• The hydrophobicity of the wild-type and mutant residue differs. • Hydrophobic interactions, either in the core of the protein or on the surface, will be lost.
1307	ATA > AAA^a^	Ile(I) > Lys(K)	Pathological	0.72 (Pathogenic)	Disease/7	5%	WDR5 WD40 repeat (blade 5,6)‐binding ligand	Beta‐Catenin Binding	• The mutation introduces a charge, this can cause repulsion of ligands or other residues with the same charge.
1317	GAA > CAA	Glu(E) > Gln(Q)	Pathological	0.41 (Pathogenic)	Disease/4	10%	Glycosaminoglycan attachment site	Beta‐Catenin Binding	• The charge of the wild-type residue will be lost, this can cause loss of interactions with other molecules or residues.

^a^- represents Novel mutations (unreported in the database); *RI* - Reliability Index

Based on the Circos plot analysis, stage III and IV tumor samples were associated with the absence(negative) of APC protein expression, whereas Y622* and Q625* mutations were associated with stage II, III and IV tumor samples. Y622* mutated and negative APC protein expressing gastric tumor samples had a high concordance with aneuploid cells (Fig. 3). Fisher's exact test exhibited a significant statistical association of Y622* and A1247T mutations with negative APC protein expression (*P* = 0.0002; 0.005), whereas, a positive protein expression (*P* = 0.0003) was observed in association with N1108S mutation. The mutated region was responsible for down-regulation through a process mediated by direct ubiquitination which will affect the protein function and alter the cell cycle regulation.

## Discussion

Mutations of APC gene has been shown to play an important role in colorectal tumorigenesis [33]. In the current study, we have found a significant relationship between the APC mutation, cell cycle regulation and protein expression indicating a positive role of the mutations in diffuse type gastric adenocarcinomas. We have found frequent pathogenic mutations at codon 622 of exon14 APC in gastric tumors that generates stop codon (Y622*). All the samples containing codon 622 mutation showed abnormal cell cycle regulation. All the samples containing 622 and 625 codon mutation coded for a truncated protein and resultant cells were aneuploid with high S phase. Tumor samples with codon 622,625 and 1307 mutations were strongly associated with the negative expression of the APC protein in cytoplasm as shown in immunohistochemistry analysis. Previous study showed that truncations in APC eliminate microtubule binding contributing to chromosome instability (the CIN phenotype) in colon cancer cells because they directly affect chromosome-spindle attachment [13]. Phosphorylation of APC by Bub kinases may be an important aspect of CIN phenotype, explaining why the loss of Bub1 kinase activity is a common feature of colon cancer cell lines [13]. Loss of APC function results in microtubule plus-end attachment defects during mitosis and consequent chromosome misalignment and CIN [34]. Due to exon 14 mutation, APC protein might be truncated and the same phenomenon might occurin gastric cancer leading to aneuploidy and G1 phase arrest followed by high S phase in cell cycle for diffuse type gastric cancer. It is evident that the two mutations in exon 14 of APC gene were independent of each other and are responsible for loss of protein function based on our data analysis and from the APC Mutation Database.

Our results are in agreement with the finding that mutations of APC in sporadic cases have been detected in coding amino acids within a short range from 1058 to 1317, which exists inside exon15 and is called the mutation cluster region (MCR) [35]. Mutations in exon 15 of APC gene were detected in 40% of gastric cancers and are similar to the previous studies [17]. Our results imply that APC plays a crucial role in gastric carcinogenesis as was observed in colorectal carcinogenesis [33]. The mutations detected were located in relatively small part of exon 15. Since exon 15 is extremely large, covering codons 654 through 2843 (77% of the whole coding region), it is probable that this exon encodes one of the important domains of the gene product. Alternatively, this region may be a hot spot of mutation for targeting by carcinogens. One particular missense variant, I1307K, is found in Tripura population, and carriers of this allele are at several fold higher risk of developing multiple gastric adenomas and colorectal cancer [36]. As the I1307K variant consist of T-A substitution producing a poly (A) tract, it was assumed that the variant precipitated polymerization error during DNA replication, and thus indirectly predisposition to cancer [36]. E1317Q codes for a mutation in the MCR region of the APC gene at β-catenin binding site and this mutation acts like I1307K, by a dominant negative effect on the APC/β-catenin pathway, thus leading to adenoma [37]. E1317Q mutation has been detected in colorectal polyps or cancer like as in the case of the present study [38].

The antisera that react with the specific epitope of the APC protein were used for immuno-histochemical staining to demonstrate the protein expression in gastric tumors. The subcellular localization of the APC protein is reported to be predominantly cytoplasmic in normal tissues, though mammary epithelium has been reported to show equal distribution of cytoplasmic and nuclear APC [25].In the present study, APC protein was detected more in the cytoplasmic staining in gastric tumors when compared to normal tissues. An antibody for the C-terminal region of the APC protein that detects only full-length or wild-type APC protein was used in the present study. The mutated APC protein loses its binding site in the β-catenin destruction complex resulting in low expression of APC in the cytoplasm and nucleus, which ultimately results in decreased membrane expression [39].The gastric cancer tumor in stage III and stage IV showed negative expression of the protein in cytoplasm and nucleus.

Sequencing analysis confirmed that the mutations in exons 14 and 15 of APC gene resulted in truncation of the gene products or in an amino acid change. APC gene encodes a large protein with multiple cellular functions and mutations in this gene lead to alterations in signal transduction, differentiation, intercellular adhesion, cytoskeletal stabilization, cell cycle and apoptosis [40, 41]. Truncating mutations in exons 14 and 15 were strongly associated with gastric and colorectal cancer [42]. A wealth of data shows that almost all colorectal tumors with APC mutations lose the SAMP (connexion/actin/β-catenin binding) repeats and all,or otherwise one or two of the seven β -catenin binding/degradation sites. Colorectal tumour retains a truncated APC protein to control the transcriptional activity of β -catenin and avoids it to reach too high levels, which is detrimental for tumour growth, in agreement with the “just right signalling” model [43]. The truncated APC can influence the transcriptional activity of β -catenin by at least two different mechanisms: a stimulation of the transcriptional activity of β -catenin upon APC downregulation without any obvious increase of the β-catenin level [44] and alternatively, truncated APC might be required for tumour development independently of its control over the transcriptional activity of β -catenin as previously discussed [45]. The APC protein might be truncated and all the regulatory features might be lost, especially the feature responsible for down-regulation through a process mediated by direct ubiquitination.

In the present study, eight APC mutations in exons 14 and 15 were all detected in diffuse type gastric cancer. The codon 622 and 625 mutations are significantly associated with cell cycle abnormality. This result indicates that APC gene is mutational target for gastric cancer tumor cells and supports the hypothesis that APC mutation-positive tumors may identify an alternative pathway which is probably different from the normal pathway. Our study showed that mutations in APC can contribute to development of diffuse type gastric adenocarcinomas by altering the APC protein expression and cell cycle regulation and additional genetic changes could account for the differences in pathology.

## Conclusion

The present study suggests the implication of novel APC gene alterations in gastric cancer related with cell cycle abnormalities and APC protein expression in diffuse type gastric cancer. Our findings need to be confirmed by a larger cohort study, however, we reduced the risk of false-positive diagnosis of patients with other diseases by enrolling the patients with only diffuse type gastric cancer.

## Additional file

Additional file 1: Figure S1. (A) PCR-RFLP of APC gene Exon 14 (M – 100 bp marker; S1, S2 – Normal alleles; S3, S4 – Mutant alleles); (B) SSCP analysis of APC gene exon 15A.b. (1, 2 – Healthy Control; 3,4,5,6 – Tumour tissues; 7, 8 – Matched tissues). **Figure S2.** Exon 15 non-synonymous mutation positions in the APC protein (DOCX 856 kb).

## Abbreviations

μl: Microliter
μm: Micro meter
APC: Adenomatous Polyposis Coli
DNA: Deoxyribonucleic acid
H: Hour
kDa: Kilo Dalton
MCR: Mutation cluster region
Min: Minute
mM: Mile Moller
PBS: Phosphate Buffer Saline
PCR: Polymerase chain reaction
RNA: Ribonucleic acid
SNP: Single Nucleotide Polymorphism
SSCP: Single stranded conformation polymorphism
Vh: Volt-hours
