# Supplementary material for: Novel APC gene mutations associated with protein alteration in diffuse type gastric cancer
Source: BMC Med Genet. 2017 Jun 2;18:61. doi: 10.1186/s12881-017-0427-2 (PMC5457612; doi:10.1186/s12881-017-0427-2)
Supplement: Additional file 1: Figure S1. — (A) PCR-RFLP of APC gene Exon 14 (M – 100 bp marker; S1, S2 – Normal alleles; S3, S4 – Mutant alleles); (B) SSCP analysis of APC gene exon 15A.b. (1, 2 – Healthy Control; 3,4,5,6 – Tumour tissues; 7, 8 – Matched tissues). Figure S2. Exon 15 non-synonymous mutation positions in the APC protein (DOCX 856 kb). [file 12881_2017_427_MOESM1_ESM.docx]

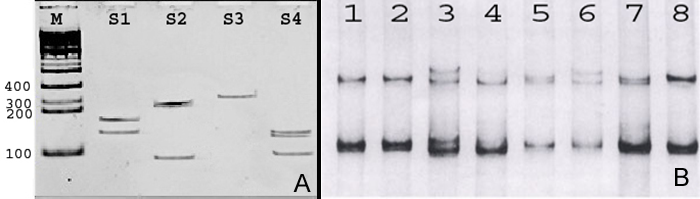


**Supplementary Fig. 1** (A) PCR-RFLP of APC gene Exon 14 (M – 100 bp marker; S1, S2 – Normal alleles; S3, S4 – Mutant alleles); (B) SSCP analysis of APC gene exon 15A.b. (1, 2 – Healthy Control; 3,4,5,6 – Tumour tissues; 7, 8 – Matched tissues).


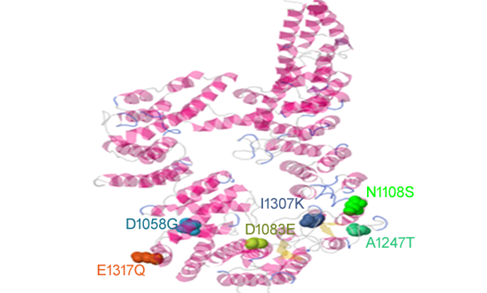


**Supplementary Fig. 2** Exon 15 non-synonymous mutation positions in the APC protein.
